# Copper Sulfide Nanoparticles-Incorporated Hyaluronic Acid Injectable Hydrogel With Enhanced Angiogenesis to Promote Wound Healing

**DOI:** 10.3389/fbioe.2020.00417

**Published:** 2020-05-08

**Authors:** Wencheng Zhou, Liu Zi, Ying Cen, Chao You, Meng Tian

**Affiliations:** ^1^Neurosurgery Research Laboratory, National Clinical Research Center for Geriatrics, West China Hospital, Sichuan University, Chengdu, China; ^2^Department of Burns and Plastic Surgery, West China Hospital, Sichuan University, Chengdu, China; ^3^Department of Integrated Traditional and Western Medicine, West China Hospital, Sichuan University, Chengdu, China; ^4^Department of Neurosurgery, West China Hospital, Sichuan University, Chengdu, China; ^5^West China Brain Research Centre, West China Hospital, Sichuan University, Chengdu, China

**Keywords:** copper sulfide, nanoparticle, hydrogel, angiogenesis, wound healing

## Abstract

Skin wound caused by trauma, inflammation, surgery, or burns remains a great challenge worldwide since there is no effective therapy available to improve its clinical outcomes. Herein, we report a copper sulfide nanoparticles-incorporated hyaluronic acid (CuS/HA) injectable hydrogel with enhanced angiogenesis to promote wound healing. The prepared hydrogel could not only be injected to the wound site but also exhibited good photothermal effect, with temperature increasing to 50°C from room temperature after 10 min of near-infrared light irradiation. The cell culture experiments also showed that the hydrogel has no cytotoxicity. In the rat skin wound model, the hydrogel treated wounds exhibited better healing performances. Masson’s trichrome staining suggested that collagen deposition in wounds treated with the hydrogel was significantly higher than other groups. The immunohistochemical staining showed that the hydrogel can effectively upregulate the expression of vascular endothelial growth factor (VEGF) in the wound area at the incipient stage of healing, and the CD 31 immunofluorescence staining confirmed the enhanced angiogenesis of the hydrogel. Taken together, the prepared CuS/HA hydrogel can effectively increase the collagen deposition, upregulate the expression of VEGF, and enhance the angiogenesis, which may contribute to promote wound healing, making it a promising for application in treating skin wound.

## Introduction

Skin wound resulting from trauma, inflammation, surgery, or burns is a common issue that needs to be treated immediately (Browne and Pandit, 2015). However, up to now, an effective wound healing remains a significant challenge due to its complex biological process where various intracellular and intercellular pathways should be activated and coordinated to accelerate and enhance the healing (Das and Baker, 2016). Among these biological processes, angiogenesis plays many important roles during the healing since the newly formed vessels are of great significance to nutrients and growth factors transport and granular tissue growth, and thus encouraging angiogenesis is regarded as one of the most essential steps for promotion of wound healing. In this regard, earlier studies mainly focused on the introduction of angiogenic growth factors such as vascular endothelial growth factor (VEGF), and basic fibroblast growth factor (bFGF). Nevertheless, the use of growth factors was limited by their high cost and side effects.

Recently, some metal ions have attracted more and more research interest due to their vascularization effect (Yu et al., 2019). For example, copper ions have been reported that could stimulate angiogenesis by secretion of VEGF and thus promote wound healing. Moreover, nano-formed copper such as copper sulfide nanoparticles (CuS NPs) is capable of photothermal therapy induced by near-infrared (NIR) light irradiation which would be effective in killing bacteria as a non-resistant and minimally invasive process (Zhou et al., 2015; Feng et al., 2019). As a result, CuS NPs may offer both angiogenesis and antibacterial ability, both of which are beneficial to accelerate wound healing (Li Z. et al., 2018; Shanmugapriya and Kang, 2019). However, the use of copper ions or CuS NPs alone is not suitable for wound healing since direct contact may produce inflammation in skin tissues and the administration will easily detach from the wound as well. Considering these shortages, it is potential to incorporate copper ions or CuS NPs into carriers for wound healing.

Hydrogel composed of cross-linked hydrophilic polymer chains has its innate merits as wound dressing or drug carrier for wound healing, resulting not only from its good water-reserving ability that is suitable for absorption of wound exudates and skin cell survival and metabolism but also from its interconnected pores that provide three-dimensional networks with large volume and surface area for the incorporation of a drug (Li S. et al., 2018). The hydrogel can be prepared by natural polymers or synthesized ones. Compared to synthesized polymers, natural ones are more biocompatible, and more importantly, they are usually derived from the extracellular matrix (ECM). For instance, hyaluronic acid (HA) is one of the major components of the ECM consisting of disaccharide units of D-glucuronic acid-N-acetylglucosamine which contains many carboxyl and hydroxyl groups that is beneficial to water conservation for the skin. Furthermore, it was found that HA also plays an important role in the process of angiogenesis, suggesting that HA is a promising candidate to prepared hydrogel for wound healing.

**FIGURE 1:**
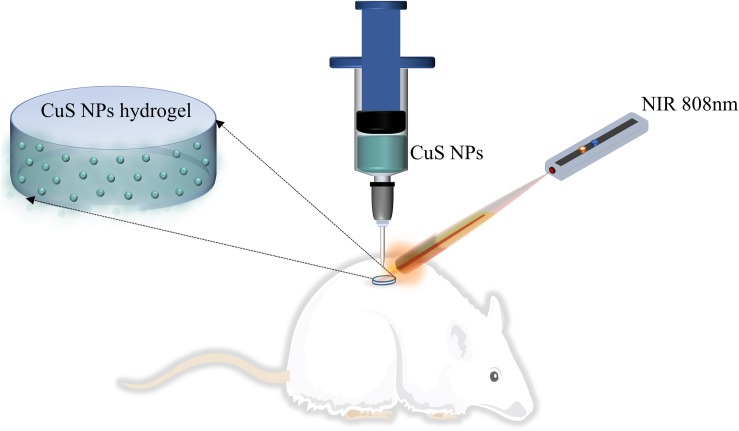
Schematic presentation of CuS NPs-incorporated HA injectable hydrogel for wound healing.

In this work, we hypothesize to prepare a CuS NPs-incorporated HA injectable hydrogel, which can not only be injected to the wound site but can also be capable of photothermal therapy induced by NIR light irradiation (as shown in Figure 1). Moreover, both the use of CuS NPs and HA may benefit to enhance angiogenesis and thus promote wound healing. To address this hypothesis, CuS NPs and thiolated HA were first synthesized, and then CuS/HA hydrogel was prepared and characterized in terms of gelation time, morphology, photothermal effect, and cytotoxicity, before the *in vivo* angiogenesis and wound healing ability was evaluated in a rat skin wound model.

## Materials and Methods

### Materials

Copper chloride (CuCl_2_), sodium sulfide (Na_2_S 9H_2_O), and sodium citrate were purchased from Kelong Co., Ltd. (Chengdu, China). Methoxy-PEG-thiol (SH-PEG, molecular weight 5000 Da) was purchased from ToYongBio Co., Ltd. (Shanghai, China). HA sodium salt from Streptococcus Equi was supplied by Aladdin Co., Ltd. (Shanghai, China) 0.5, 5′-Dithiobis (2-nitrobenzoic acid; DTNB), N-(3-Dimethylaminopropyl)-N’-ethyl carbodiimide hydrochloride (EDAC), cysteamine, and dithiothreitol (DTT) were purchased from Sigma (St. Louis, MO, United States). N-hydroxysuccinimide (NHS) was obtained from Pierce. Isoflurane was obtained from RWD Life Science Co., Ltd. (Shenzhen, China). Cell counting kit-8 (CCK-8) was obtained from Dongren Chemical Technology Co., Ltd. (Shanghai, China). Hematoxylin and eosin (H&E) stains and Masson’s Trichrome Stain Kit were purchased from Beijing Solarbio Science & Technology Co., Ltd. (Beijing, China). The antibody of endothelial growth factor anti-VEGF was bought from Proteintech (Chicago, United States). The anti-CD31 antibody was from Affinity Biosciences, Inc. (Cincinnati, OH, United States). Deionized water (18 MΩ) was obtained from a Milli-Qsynthesis system (Millipore, Billerica, MA, United States). All the above reagents are used directly without further purification.

### Synthesis and Characterization of CuS Nanoparticles (NPs)

The synthesis of CuS NPs was carried out as a facile hydrothermal route as follows (Zhou et al., 2010). Briefly, 10 mL of sodium citrate (1.0 mg mL^–1^), and 10 mL of CuCl_2_⋅2H_2_O (0.85 mg mL^–1^) aqueous solutions were added into 30 mL of ultrapure water in sequence. The whole solution was protected by argon and stirred for 30 min at room temperature. After that, dropwise added 50 μL of Na_2_S⋅9H_2_O (78 mg mL^–1^) aqueous solution into the reaction and kept on stirring another 5 min. In this process, the color of the reaction solution gradually changed from light blue to light yellow, orange, and finally to dark brown. Next, the reaction was transferred to a 90°C oil bath to react for 15 min to form green-colored CuS-citrate NPs. The whole mixture was moved to ice-cold water. To obtain PEG-coated NPs, 1 mg of SH-PEG was added to the 1 mL CuS-citrate NPs solution (200 μgmL^–1^) to introduce the PEG coating. The reaction was performed overnight at room temperature to obtain PEG-coated CuS NPs.

The morphology of the CuS NPs was observed by transmission electron microscopy (TEM) performed on a Hitachi HT7700 (Japan). Specimens were prepared by adding 50 μL micellar solutions onto a copper grid followed by staining with phosphotungstic acid (1 wt%) for 1 min and then dried with filter paper. The size, zeta potential, and polymer dispersity index (PDI) of the CuS NPs were determined by dynamic light scattering (DLS) on a NanoBrook Series Particle/Protein Size and Zeta Potential Analyzer.

### Synthesis and Characterization of Thiolated HA

The synthesis of thiolated HA is illustrated in Supplementary Figure S1A. The thiolated HA was synthesized by the coupling of cysteamine onto the HA molecular chains through EDC chemistry. Briefly, 1 g of HA sodium salt was dissolved in 200 ml distilled water, and then cysteamine and EDAC were added as solids to the reaction with a molar ratio of -COOH/cysteamine/EDAC 1:2:2. The pH of the reaction solution was maintained at 4.75 by the addition of 1 M HCl. After 4 h the reaction was stopped by neutralizing the solution by addition of 4 M NaOH. The solution was dialyzed (3500 cutoffs) against distilled water for 3 days at room temperature. 5 *g* DTT was then added to the resulting solution and the pH of the solution adjusted to 8.5. After stirring for 8 h under N_2_, the pH of the solution was adjusted to 4.0 by the addition of 1 M HCl. The resulting solution was first dialyzed (3500 cutoffs) against HCl solution (pH 4.0) containing 100 mM NaCl under N_2_, followed by dialysis (8000 cutoffs) against HCl solution (pH 4.0) under N_2_. The solution was clarified by centrifugation, and the supernatant was sterilized with a 0.2 μm Millipore filter and then lyophilized. The product was stored at −20°C and protected under N_2_. The degree of substitution (DS) of free thiols was determined using the Ellman method (Shu et al., 2002). The structure of thiolated hyaluronan was characterized by 1H NMR spectrum in D_2_O.

### Preparation of the Injectable CuS/HA Hydrogel

The CuS/HA hydrogel was prepared by simply mixing with thiolated HA solution and CuS NPs solution to obtain the precursor gelation solution, and then the gelation was initiated with the hydrogen peroxide solution. Gelation time was determined by a test tube inverting method. The final concentration of thiolated HA, CuS NPs, and hydrogen peroxide was 2% w/v, 200 μg/ml, and 0.03% v/v, respectively. This injectable CuS/HA hydrogel was named G II. The blank hydrogel without CuS NPs was used as a control (named G I). Scanning electron microscopy (SEM) was used to observe the morphology of the freeze-dried hydrogel. After coating with gold, the cross-sectional morphology was viewed with a ZEISS EVO 10 microscope. The storage and loss modulus were measured with a plate-to-plate rheometer (MCR 302, Anton Paar, Ashland, VA, United States) using a 25 mm plate under a constant strain of 1% and frequency of 10 rad/s.

### *In vitro* Photothermal Effect

The photothermal effect of CuS NPs was carried out in two parts, nanoparticles solution, and hydrogels. For the solution, different concentrations (200, 100, 50, 20, and 10 μg/mL) were exposed to 808 nm laser at 1 W/cm^2^ for 10 min and the temperatures were recorded every other min interval by an infrared imaging camera (FLIR ONE PRO, United States). To further test the stability of the photothermal effect, 200 μg/mL CuS NPs were determined to heat by the laser in the same condition and then cool down naturally for several cycles. On the other hand, the photothermal effect of CuS/HA hydrogels was evaluated in the same approach as above.

### Cell Experiments

Mouse embryonic fibroblast (NIH/3T3) cell lines were chosen to perform the cell experiments. The cells were purchased from the ATCC and cultured in Dulbecco’s modified Eagle’s medium (DMEM) supplemented with 10% fetal bovine serum (FBS; Gibco, United States), 100 mg/ml penicillin, and 100 mg/ml streptomycin. The cells were grown at 37°C in an atmosphere of 95% humidified air containing 5% CO_2_. Experiments were carried out when cells were in the logarithmic phase of growth. Cytotoxicity is a classic problem for biomaterials. According to ISO10993 part 5 guidelines 3, CCK-8 was used to examine the cytotoxic effects of the hydrogel, and solution of CuS NPs. For the test of CuS NPs solution, samples of three concentrations were used for testing (200, 100, and 50 μg/ml). For the injectable hydrogel test, both GI and GII were included. The extracts of these samples were prepared by the incubation of each sample with 1 ml of DMEM supplemented with 10% FBS for 48 h at 37°C.3T3 cells were cultured with common conditions [DMEM supplemented with penicillin (100 mg/ml), streptomycin (100 mg/ml), and 10% FBS, 37°C, 5% CO_2_] until they reached approximately 80% confluency before cytotoxicity assay. After that, the cells were seeded into 96-well plates at a density of 3000 cells/well. After another 24 h incubation, the cells cultured in medium without test samples were used as a control group and the culture media of other wells were discarded and replaced with the medium containing the three concentration solutions of samples or two extracts (12.5%, v/v). After further cultured for designed time (0, 24, 48, and 72 h), the cells were subjected to cell toxicity assays by CCK-8. Briefly, the cells washed by phosphate buffer saline (PBS) and were incubated in a fresh culture medium containing 10% CCK-8 to incubate for 3 haway from light. Finally, the supernatant was transferred and the absorbance of the solution was determined at a test wavelength of 450 nm. The ratio of the optical density (OD) of the test samples to that of the control group presented the cell proliferation rate.

### Cutaneous Wound Healing Experiments

The wound healing experiment *in vivo* for samples was conducted on Sprague–Dawley male rats (SD rats). All the animal experiments and procedures were approved by the Animal Ethical Committee of the West China Hospital of Sichuan University in compliance with Chinese national guidelines for the care and use of laboratory animals. Eight weeks old SD male rats (200 ∼ 220 *g*) were purchased from Da-Shuo Laboratory Animal Co., Ltd. (Chengdu, China). The rats were kept under controlled temperature and humidity with food and water *ad libitum*. After standard anesthetization with anesthetic ventilator using isoflurane (4%for induction in an induction chamber, and 2% for maintenance), the back of each animal was shaved to expose the injection site. A skin biopsy apparatus (6 mm) was used to make the round section of the full-thickness skin injury on the back of the rat skin. All procedures were performed under aseptic conditions during the surgery process. There were six round wounds (6 mm in diameter) created on the back of each rat and 7 rats were included, so there were 30 wounds served as experimental subjects, and 12 wounds as back-ups in case of experimental animal death. After the removal of wound skin, 100 μL of different injectable hydrogels were synthesized *in situ* (methods as mentioned before) 0.30 wounds were randomly divided into 6 groups (I-VI). Group I was the blank control group with nothing dressed after a skin injury, group II wounds received NIR radiation after the operation, group III wounds were dressed with the hydrogel G II, wounds in group IV were dressed with the hydrogel G II and treated with NIR, group V wounds were dressed with the hydrogel G I, and group VI wounds were dressed with the hydrogel G I and treated with NIR. For NIR treated groups, the wound area was irradiated with an 808 nm NIR laser (1 W/cm^2^) for 10 min after the operation 10 min, 2 days, and 3 days and infrared photos were taken at the same time. The gross image of the wounds was taken after the operation on days 0, 3, 7, 10, and 14. To monitor the wound healing process, ImageJ software was employed to process the images to obtain the wound area. The healing rate (HR) was calculated, according to the formula as below:

$$H\,R=\frac{\left(S_{0}-S_{\text{x}}\right)}{S_{0}}\times 100\%$$

where *S*_0_ was the initial area on day 0 and *S*_x_ represented the wound areas on days 3, 7, 10, and 14, respectively.

### Hematoxylin-Eosin and Masson’s Trichrome Staining

Rats were sacrificed on the 3rd, 7th, and 14th days after the operation, respectively, and the skin tissue (1 × 1 cm^2^) containing the wound was taken for dermatological examination. The samples were fixed in 4% paraformaldehyde, dehydrated in graded alcohol, and embedded in paraffin. The tissue blocks were sectioned into 5 μm thick slices. The sections were stained via H&E and Masson’s trichrome to carry on histological analysis and collagen formation assessment. 8 All photos were captured by a microscope (BX43, Olympus, Japan). Histopathological scores of HE slices were employed to a semiquantitative evaluation of wound healing according to the criterion of Eldad et al. (1998). Collagen volume fraction (CVF) was utilized to assess collagen formation (Singh et al., 2019). Specifically, 6 regions of interest (ROIs) per HPF were recorded under a microscope. Then, the CVF of each ROI was assessed using the ImageJ software to semiquantitative analysis collagen content.

### Immunohistochemical and Immunofluorescence Staining

Angiogenic vessel markers CD31 and VEGF were chosen in our experiment because they play an important role in angiogenesis (Nowak-Sliwinska et al., 2018). Immunofluorescence and immunohistochemical staining of CD31 and VEGF were carried out as previous literature. The rehydrated skin tissue sections were boiled in sodium citrate buffer for about 20 min before anti-CD31 and anti-VEGF (vascular endothelial growth factor) antibody incubated the sections overnight at 4°C, respectively. For CD31, DAPI stained the nuclei. The distribution of CD31 was observed by a fluorescence microscope and vascular density (VD) was expressed as the percentage of positive CD31 staining per ROI which were calculated using ImageJ software (Du et al., 2017). For VEGF, hematoxylin counterstained the nuclei and the integral optical density (IOD) of each view was assessed using the ImageJ software to determine its content (Zhou et al., 2020).

### Statistical Analysis

The experimental data are expressed as mean ± standard deviation. Multiple comparisons among groups were determined using two-way ANOVA followed by Tukey’s multiple comparisons test with adjusted *P* value. Significance was presented as: ^****^for *P* < 0.0001, ^∗∗∗^for *P* < 0.001, ^∗∗^for *P* < 0.01, and ^∗^for *P* < 0.05.

## Results and Discussion

### Synthesis and Characterization of CuS NPs

**FIGURE 2:**
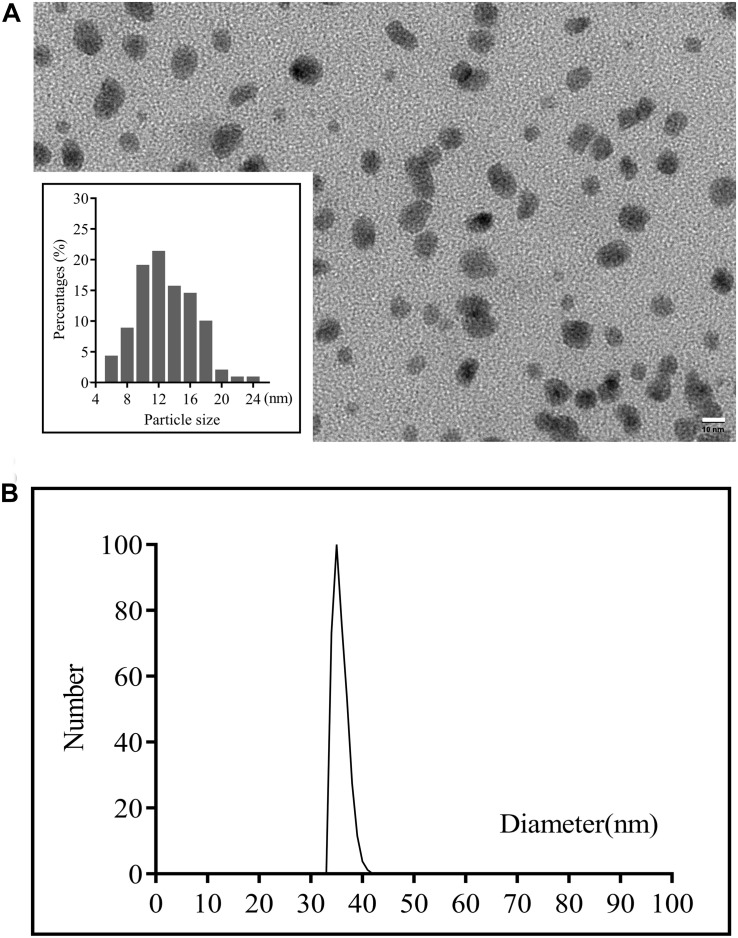
**(A)** TEM images of the CuS NPs (scale bar 50 nm) and **(B)** Particle sizes of the CuS NPs detected by DLS.

The CuS NPs were synthesized by a simple one-pot approach where CuS-citrate NPs were first prepared by reacting CuCl_2_ and Na_2_S in the presence of sodium citrate, and then PEG coating was introduced by incubation of CuS-citrate NPs with thiolated-PEG to disperse the NPs in the solution and incorporate into the hydrogel uniformly. The morphology of the synthesized CuS NPs was confirmed by TEM showing in Figure 2A where the NPs were in good dispersion and uniform size, with an average diameter of 12 nm. This diameter is of great significance for drug delivery through the blood vessels (Ding et al., 2019). The hydrodynamic average diameter of the CuS NPs was 35 nm as shown in Figure 2B determined by DLS. The Zeta-potential and PDI of the NPs was -6.07 mV, and 0.219, respectively. The negative charge of the NPs was probably due to the PEG layer, which is consistent with a previous report (Lin et al., 2019).

### Synthesis and Characterization of Thiolated HA

Thiolated HA was synthesized using an amide condensation reaction between HA and cysteamine in the presence of EDC and followed by reduced using DTT. As shown in Supporting Information Supplementary Figure S1, the chemical structure of the final product was confirmed by 1H NMR spectrum in D_2_O where two new peaks have appeared, one is at 2.8 ppm corresponding to the hydrogen of methylene close to thiol, and the other is at 2.6 ppm that assigned to the hydrogen of methylene adjacent to amide, suggesting that the Thiolated HA was successfully synthesized. The content of thiol was 0.49 mmol/g corresponding to 40% of the substitution degree as determined by the Ellman method.

### Preparation of the Injectable CuS/HA Hydrogel

**FIGURE 3:**
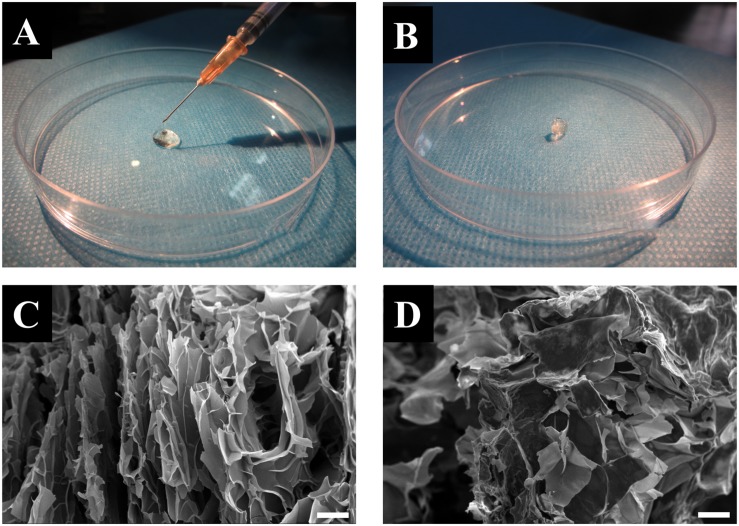
**(A)** Injection operation of the CuS/HA hydrogel before gelation. **(B)** The gelated CuS/HA hydrogel. **(C)** SEM image of a cross-section of CuS/HA hydrogel, scale bar 100 μm. **(D)** SEM image of a cross-section of HA hydrogel, scale bar 100 μm.

Both the CuS/HA hydrogel and blank hydrogel were formed by crosslinking with the disulfide bonds that initiate by oxidization of the thiolated groups along the HA chains with hydrogen peroxide. The gelation time of the CuS/HA and blank hydrogel was similar, approximately 6 min. Within this gelation time, the hydrogel could be injected into the skin wounds. Figures 3A,B show the injection operation of the CuS/HA hydrogel before gelation and the gelated CuS/HA hydrogel, respectively. The SEM images of a cross-section of CuS/HA hydrogel and blank hydrogel were shown in Figures 3C,D, both of which exhibited interconnected pores that provide three-dimensional networks with large volume and surface area. The storage (G’) and loss modulus (G”) were measured with a plate-to-plate rheometer. As shown in Supplementary Figure S2, the G’ for HA hydrogel was 1820 Pa, while the one for CuS/HA hydrogel was 1930 Pa, and indicating that the hydrogel containing CuS NPs exhibited a higher modulus.

### *In vitro* Photothermal Effect

**FIGURE 4:**
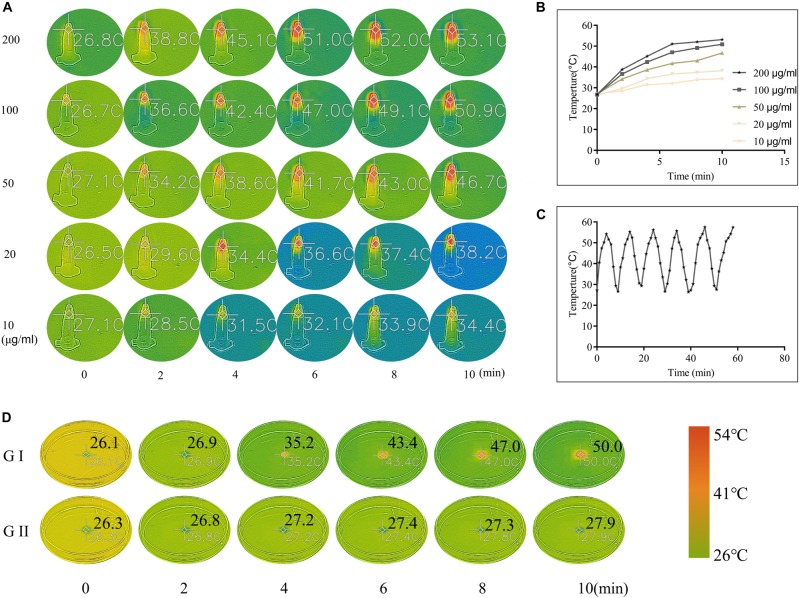
**(A,B)** Photothermal images and corresponding heating curves of CuS NPs with different concentrations upon 808 nm laser exposure. **(C)** The heating curve of the CuS NPs upon repeated 808 nm irradiation and followed natural cooling. **(D)** Photothermal images of CuS/HA (GI) and blank hydrogels (GII) upon 808 nm laser exposure. (**A** and **D** share the same color scale).

The *in vitro* photothermal effect of the CuS NPs and CuS/HA hydrogel was studied to confirm their photothermal conversion. As shown in Figures 4A,B, the CuS NPs with different concentration (10, 20, 50, 100, and 200 μg/ml) was irradiated with NIR light at 808 nm and the results showed that the CuS NPs exhibited a significant increase in temperature upon 808 nm irradiation at all concentrations. In particular, the increase in temperature was dependent on the concentration of the CuS NPs, with the final temperature of the five concentrations reaching 34.4, 38.2, 46.7, 50.9, and 53.1°C after 10 min exposure, respectively, indicating that the synthesized CuS NPs has a good photothermal effect. The photothermal stability of the synthesized CuS NPs was also studied. As shown in Figure 4C, the heating curve of the CuS NPs of each cycle was identical upon repeated irradiation, with temperature from 27 to 53°C, and there was no significant change after repeated laser exposure. These results suggested that the synthesized CuS NPs displayed good photothermal stability. The photothermal images of CuS/HA and blank hydrogels upon 808 nm laser exposure were shown in Figure 4D. Like NPs, the CuS/HA hydrogel also exhibited good photothermal effect, with increasing temperature of 50°C after 10 min exposure. In contrast, there was no increase in temperature for blank hydrogel due to without CuS NPs.

### Cytotoxicity

**FIGURE 5:**
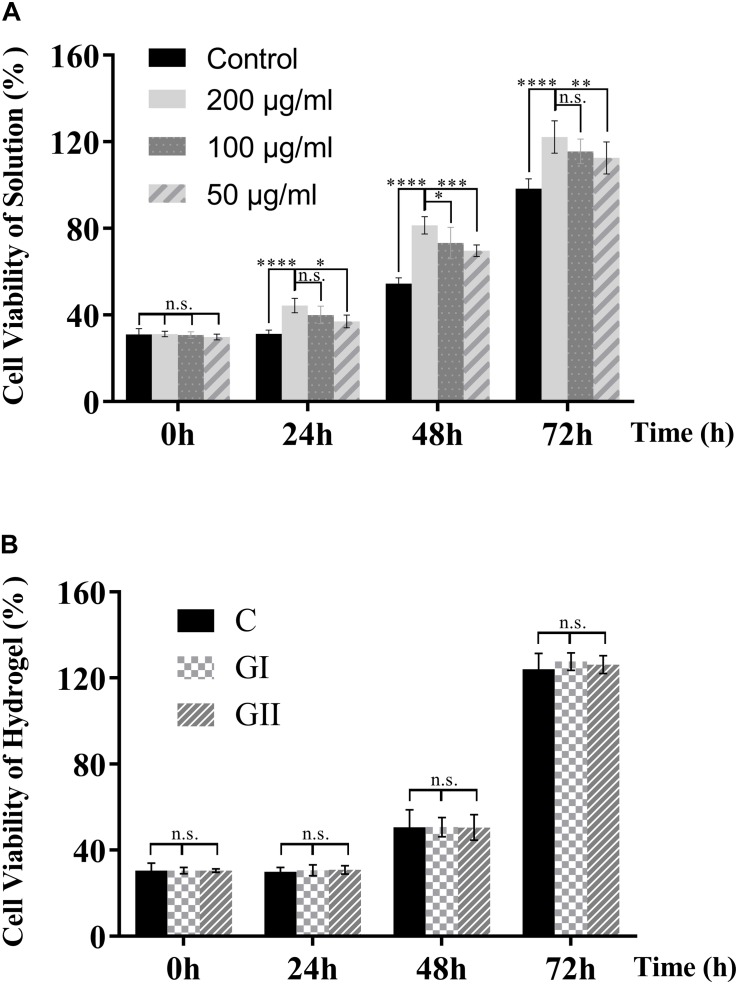
Cytotoxicity of the CuS NPs and CuS/HA hydrogel. **(A)** The cytotoxicity of the CuS NPs solutions with different concentrations. **(B)** The cytotoxicity of the hydrogels (G I and G II) compared with the blank control. Error bars represent means ± SD for *n* = 5, ****for *P* < 0.0001, ***for *P* < 0.001, ** for*P* < 0.01, and *for *P* < 0.05.

The evaluation of biocompatibility such as cytotoxicity is the primary problem in the application of biomaterials before further experiments that can only be carried out when the material is proved to be non-toxic (Savoji et al., 2018). Herein, we first tested the cytotoxicity of the CuS NPs and CuS/HA hydrogel. As shown in Figure 5A, the CuS NPs with different concentrations show not only hardly any toxicity to the cells, but also promoting the proliferation of cells with the culture time prolonged. Cell viability shows significantly CuS NPs concentration dependency, e.g., the higher the NPs concentration, the better the cell growth. In particular, the NPs with a concentration of 200 μg/ml can significantly promote cell proliferation during the whole test. The mechanism underlying the promotion of the proliferation of the cells has been suggested to relate with them directly or indirectly stimulation effects of the copper ions (Li M. et al., 2018). For example, the copper ions stimulated the cells to secrete growth factors such as bFGF, which is known to promote the proliferation of the cells (Gopal et al., 2014). Figure 5B demonstrates that both hydrogels do not have toxicity to cells too. The above results indicated that the CuS NPs could be biosafe for the following experiment and application.

### Animal Experiments

**FIGURE 6:**
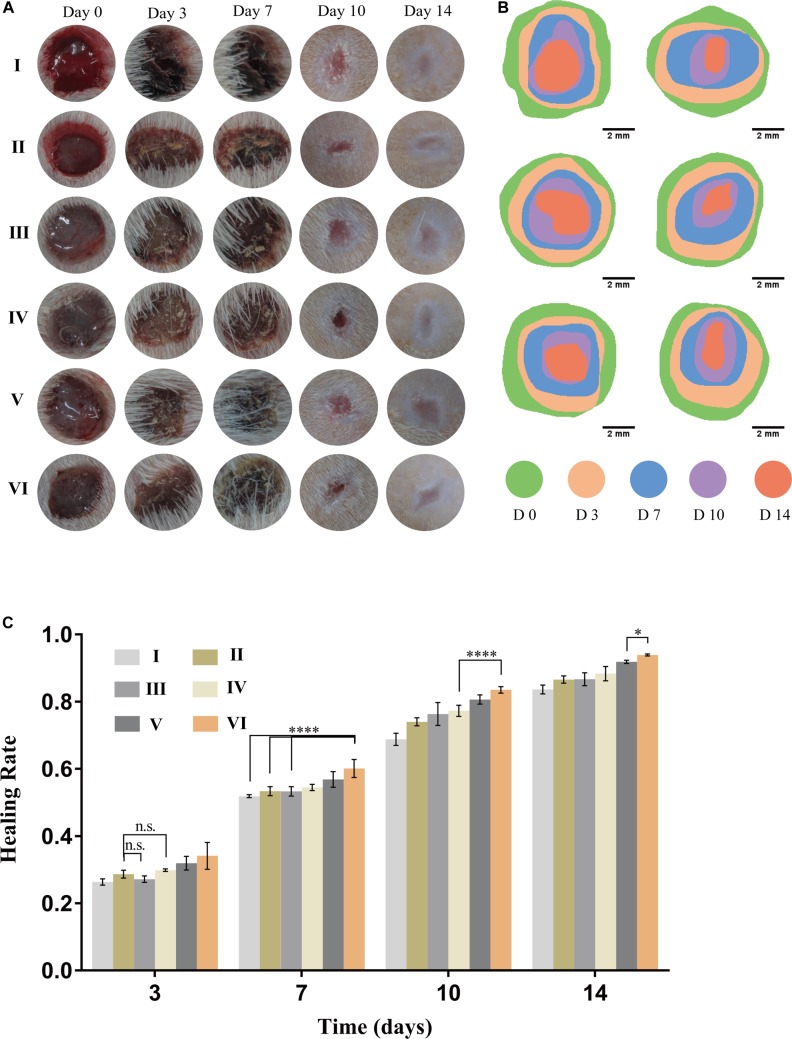
Performance of hydrogels on wound healing. **(A)** Representative gross views of wounds at predetermined time points. **(B)** Traces of wound closure area for 14 days. **(C)** Healing rate for each group, *n* = 5, data = mean ± SD. (I: blank control; II: NIR; III: HA hydrogel; IV: HA hydrogel + NIR; V: CuS/HA hydrogel; and VI: CuS/HA hydrogel + NIR).

To evaluate the effects of CuS/HA injectable hydrogel on the wound healing process, a full-thickness skin wound model was used. As shown in Figures 6A,B, in general, the wound area of all groups decreased with the prolongation of the healing time. On day 0, immediately after injection the hydrogel onto the wounds, the hydrogel can effectively stop bleeding. On day 3, compared with the blank control group I, the wounds were going to form a scar in all the other groups, and while the edema and exudation in group I were observed. By the 7th day, the difference in the healing area in each group was gradually obvious. On the 10th day, to observe and measure the area of wound healing, the hair around the rat’s wound was shaved off and we could perceive that the scars in the groups without NIR irradiated were smaller than those in the corresponding control groups. At the end of the experiment (Day 14), it is easy to notice that the groups with NIR treated present better recovery outcomes than their counterparts, not only from the smaller areas of the scars but also the lighter degree of pigmentation. Consistently, the HR for each group was shown in Figure 6C. To evaluate the safety of the photothermal effect *in vivo*, the temperature change on the wound site was also recorded. As shown in Supplementary Figure S3 the temperature of CuS/HA injectable hydrogel in the wound site changed from 32.4 to 50.3°C. On the other hand, the temperature of HA injectable hydrogel changed from 34.2 to 37.7°C. The time-temperature relationship determines the burn injury. It has been reported that burn injury occurs when the temperature reaches 50°C and the time need at least 60 min. The treated time of the highest temperature is shorter than 10 min in our experiment. Thus, the treatment is safe for animals (Martin and Falder, 2017).

### Pathomorphological Evaluation

**FIGURE 7:**
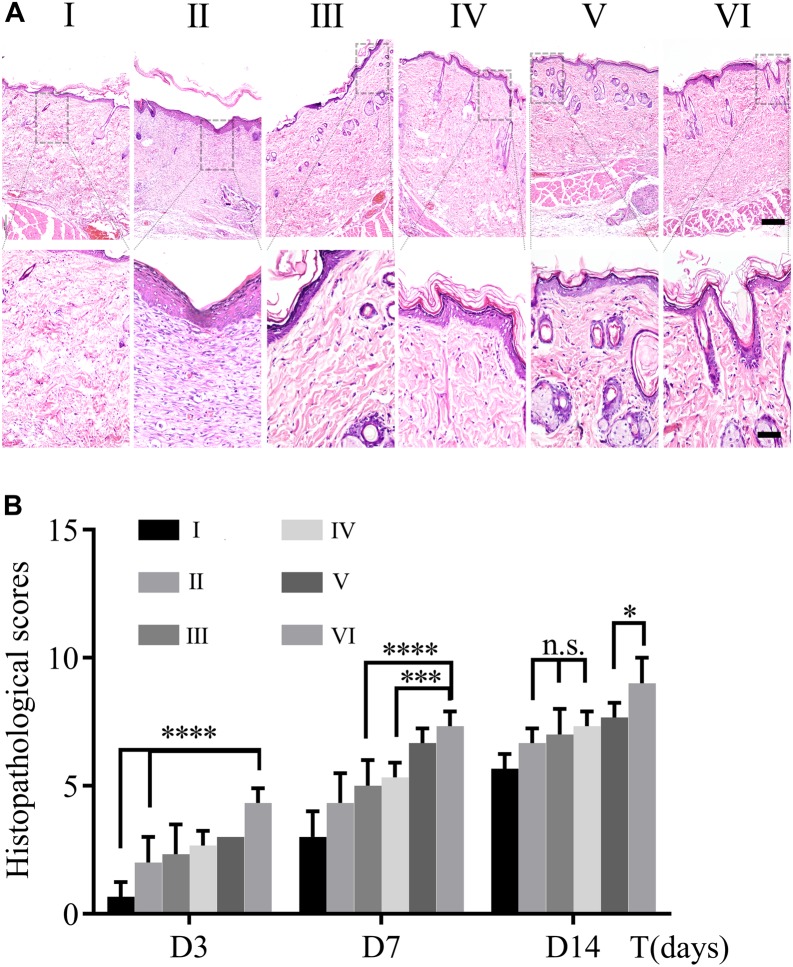
**(A)** HE staining of wound section on day 14. The scale bar of the upper panel is 200 μm; nether, 50 μm. **(B)** Histopathological scores were calculated by HE staining on day 3, 7, and 14. (*n* = 3, data = mean ± SD.) (I: blank control; II: NIR; III: HA hydrogel; IV: HA hydrogel + NIR; V: CuS/HA hydrogel; and VI: CuS/HA hydrogel + NIR).

The process of wound healing was further evaluated on the pathological level through HE staining. Figure 7A demonstrated pathological changes of skin in each group on day 14 post-operation. The skin structure was formed without a residual wound in all groups. For groups I, III, and V without NIR treated, the thickness of epidermal was thin and there were few layers of epidermal cells. They all shared these commonalities: edematous bundles of collagen, no obvious proliferation, more lasting inflammatory cells infiltration in the wound, slow formation of granulation tissue, and less neovascularization. On the contrary, the groupII, IV, and Vexposed to NIR exhibited better healing performances, e.g., cell proliferation was obvious; epidermis and dermis were thickened to a certain extent; clear epidermis cell structure was seen; inflammatory cells were reduced; necrotic tissue fell off, and cells were found to crawl. Especially in group VI, most of the wound epithelium was completely covered; there was more neovascularization; granulation tissue was thicker and collagen was more and arranged in order. To semiquantitative evaluation of wound healing, histopathological scores of HE slices were calculated based on the research of Eldad et al. As shown in Figure 7B, the scores of all groups were calculated on days 3,7, and 14, respectively. The results were following the wound healing tendency that all groups were going to heal with different degrees. It is intriguing to notice that there are differences between groups V and VI, but there is no statistical significance discrepancy between groups III and IV. Therefore, it indicates that NIR promotes wound healing on account of CuS/HA hydrogel. Furthermore, it is uncalled for to discuss the groups without NIR treated in the following tests.

**FIGURE 8:**
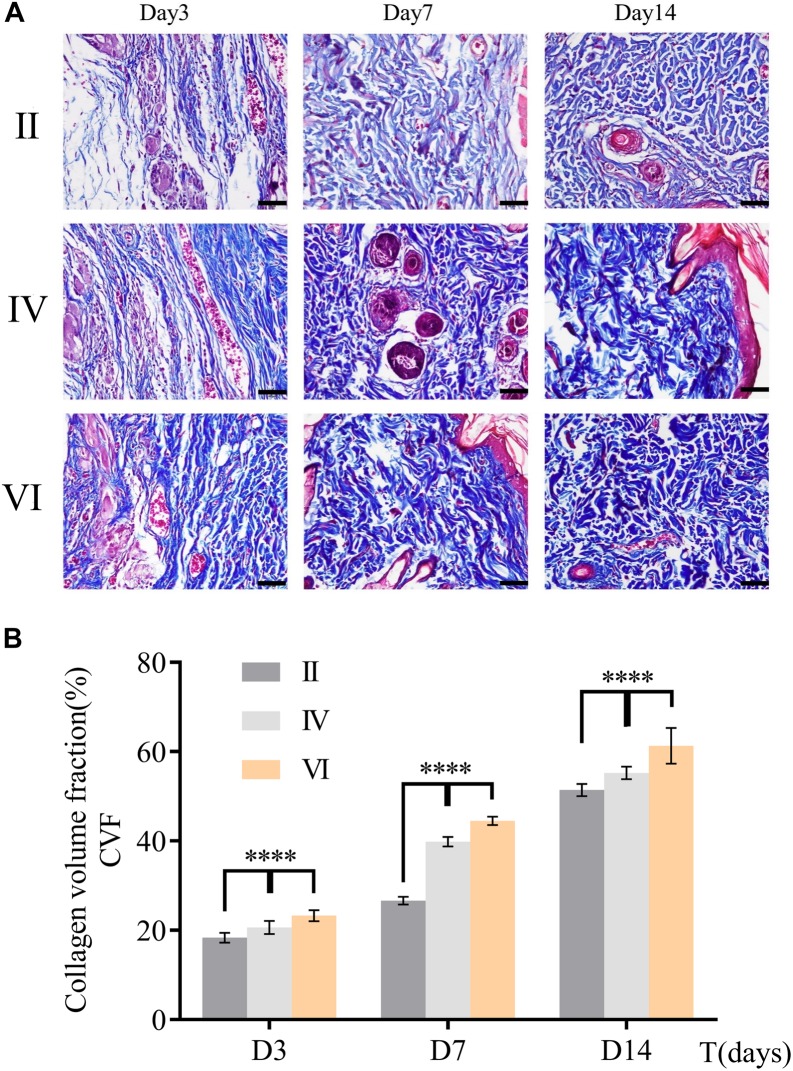
**(A)** Masson’s trichrome staining of skin sample in group B, D, and F during wound healing at different days (3, 7, and 14). Scale bar = 200 μm. **(B)** Collagen volume fraction was calculated by ImageJ. (*n* = 3, data = mean ± SD.) (II: NIR; IV: HA hydrogel + NIR; and VI: CuS/HA hydrogel + NIR).

Collagen deposition played an important role in the course of repair (Yen et al., 2018). It can be a temporary scaffold and a bed enabling migration of epithelium, keratinocytes, and microvasculature. Thus, collagen deposition was chosen as an indirect indicator to reflect the wound healing. More collagen deposition would promote wound repair in a better way and CVF would be on behalf of the collagen deposition. To determine the formation of collagen, Masson’s trichrome staining was used in this study. As depicted in Figure 8A, collagen was dyed blue in all groups. CVF was calculated using ImageJ to the semiquantitative analysis of collagen content. As shown in Figure 8B, collagen deposition in group VI had been significantly higher than other groups, even though all groups were growing. This trend consistent with HE results further clued that CuS/HA hydrogel might accelerate wound healing through increasing collagen deposition.

### Angiogenic Evaluation

**FIGURE 9:**
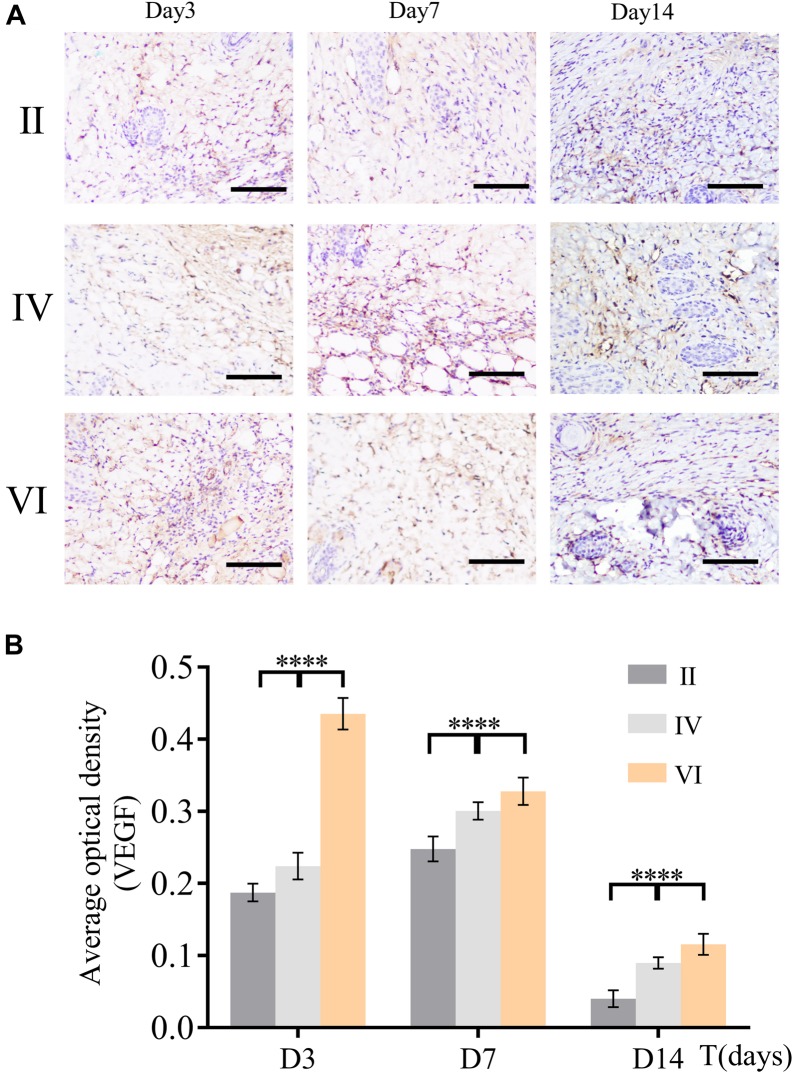
**(A)** Immunohistochemical staining for angiogenic factor VEGF of the wound tissues during the healing at different days (3, 7, and 14). Scale bar, 200 μm. **(B)** The average optional density was calculated by ImageJ. (*n* = 6, data = mean ± SD.) (II: NIR; IV: HA hydrogel + NIR; and VI: CuS/HA hydrogel + NIR).

Vascular endothelial growth factor, a specific growth factor, can bind to specific receptors on the surface of endothelial cells, which effectively stimulates the mitosis of cells and make them proliferate. Additionally, VEGF acts a pivotal part in stimulating the formation of granulation tissue and epithelium, keratinocytes, and fibroblasts, and promotes the healing of the wound. Therefore, VEGF was employed to unveil the truth of wound healing at a molecular level. As shown in Figure 9A, the skin samples of the groups treated NIR were dyed by immunohistochemical staining at predesigned day (3, 7, and 14). Further statistical analysis results in Figure 9B revealed that the tendencies among the three groups were different. Specifically, the expression of VEGF in group VI reached the peak value at the beginning (the 3rd day), then it weakened gradually over time. However, for groups II and IV, they both gradually increased from less to the maximum and then decreased. In particular, the expression of VEGF in group II and IV had never outstripped group VI from beginning to end, even though all of them hitting bottom on the 14th day. These observations indicate that CuS/HA hydrogel can effectively upregulate the expression of VEGF in the wound area at the incipient stage of wound healing to promote the formation of new blood vessels.

**FIGURE 10:**
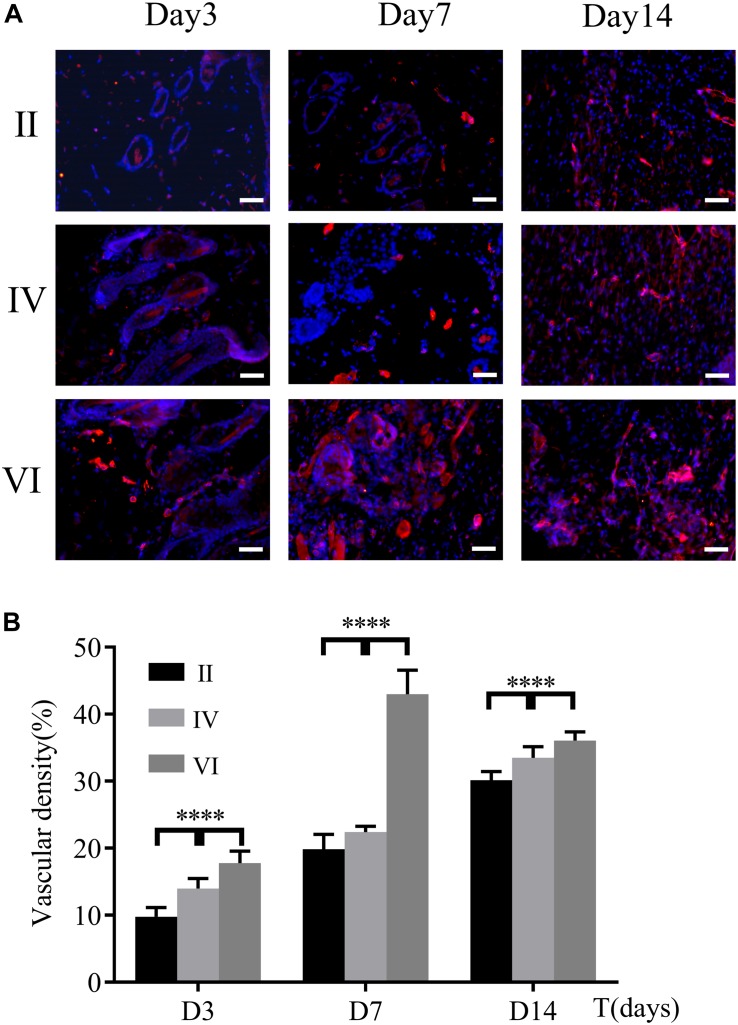
**(A)** Immunofluorescence staining for CD31 of the wound tissues during the healing on days 3, 7, and 14, respectively. Scale bar, 200 μm. **(B)** Vascular density was calculated by ImageJ. (*n* = 6, data = mean ± SD.) (II: NIR; IV: HA hydrogel + NIR; and VI: CuS/HA hydrogel + NIR).

Wound healing requires enough nutrients and metabolism for repair and reconstruction, and neovascularization is the premise and basis for the transportation of these ingredients (Burgoyne and Morgan, 2003). As a biomarker of endothelial cells, the expression of CD31 was used to evaluate the neovascularization of the wounds. As shown in Figure 10A, immunofluorescence staining of CD31 confirmed the number of newly formed blood vessels in the wounds of different groups. The expression of CD31 in group VI was always higher than the other groups and reached a peak on the 7th day (Figure 10B). However, for groups II and IV, CD31 gradually increased and finally hit the maximum on the 14th day. These observations indicated that the CuS/HA hydrogel enhanced the angiogenesis of the wound at the early stage of wound healing.

To figure out whether the VEGF expression was induced by PTT or not, we compared the VEGF expression in groups I, II, V, and VI. As shown in Supplementary Figure S4, there was a relatively low expression of VEGF in groups I and II. In contrast, both the PTT and copper ions released from CuS NPs can upregulate the VEGF expression, and the VEGF expression induced by PTT was significantly higher than that of copper ions release alone.

## Discussion

Wound healing refers to the process of healing after disconnection or defect of skin and other tissues caused by external forces on the body, including regeneration of various tissues, hyperplasia of granulation tissues and complex combination of scar formation, and showing the synergistic effect of various processes. Among them, granulation tissue has an important function of anti-infection to protect the wound and filling the gap of tissue defects (Suarato et al., 2018). Especially, angiogenesis is the critical process to form granulation tissue, because newly formed blood vessels, like a farm irrigation system, and can provide nutrients and oxygen to cells in the wound site. Therefore, promoting angiogenesis can be used as a strategy to promote wound healing.

In our study, we have found that the CuS/HA hydrogel can promote the secretion of VEGF at the beginning of the healing process. This time is pivotal for the angiogenesis, while VEGF in the other groups does not increase rapidly and sensitively. As time went on, the VEGF in the other groups then increased gradually, but they might miss the golden window phase. In the initial stage of healing, the more angiogenesis appears, the faster wound healing will be. However, the increased expression of angiogenesis in the middle and later stages is not beneficial to epidermal remodeling for the healing. The wound healing process generally consists of several intersecting and fused parts, including thrombosis, inflammation, hyperplasia, and remodeling (Wang et al., 2019). Each part is not independent, but a complex whole of intersecting and interacting with each other. The cellular proliferation status, granulation tissue formation, collagen matrix deposition, and remodeling processes are all making great contributions to efficient wound healing. Our CuS/HA hydrogels accelerate the wound healing not only by angiogenesis but also through boosting collagen matrix deposition which was proved by the observation of Masson’s trichrome staining. Why could our CuS/HA hydrogels have this talent to kill two birds with one stone? These good characteristics should attribute the success to the biological effect of copper. Copper ion is involved in the activity of many transcription factors and combines with the release complex of the cell membrane to promote the release of growth factors and cytokines. Moreover, copper can stimulate angiogenesis and collagen deposition at the same time, which has been proved by Gérard et al. (2010).

As a wound dressing, the long-term stability of CuS NPs *in vivo* should be considered. When ingested by the organism, what is the fate of NPs? All forms [zero-valent state (Cu0), ionic copper (Cu1 + orCu2 +), copper NPs] of copper may cause varying degrees of biotoxicity at high exposure concentrations (Ameh and Sayes, 2019). Some studies have shown that copper NPs have cytotoxic and genotoxic effects on human skin epidermal cells (HaCaT), which is mediated by mitochondrial pathways triggered by reactive oxygen species (ROS; Alarifi et al., 2016). Because the size of copper NPs is very small, a single copper nanoparticle can be transferred through intercellular or permeated through the cell membrane. Eventually, the particles enter the bloodstream. Most copper nanoparticles were mainly found in feces. This suggests that the colon removes most of the unabsorbed particles (Lee et al., 2016).

To make sure the needs of copper can meet while minimizing adverse effects associated with defects or overages, organisms have developed a series of strategies to maintain homeostasis. As for mammalians, they can make the Cu change not significantly through intestinal absorption, biliary excretion, and intrahepatic storage, when the organisms expose in mild to moderate concentration of Cu (Gaetke et al., 2014). These mechanisms have significant meanings in exploring the appropriate concentration of copper nanoparticles in the process of fabricating Cu NPs contained biomaterials. In our study, there was no obvious toxicity appearance, indicating that the concentration of Cu NPs used might be safe in our condition.

## Conclusion

In conclusion, we successfully prepared CuS/HA hydrogel for wound healing. Within the gelation time, the hydrogel could be injected into the skin wounds. The hydrogel exhibited good photothermal effect, with increasing temperature of 50°C after 10 min irradiation with NIR light at 808 nm, as well as no toxicity to the cells *in vitro*. In the rat skin wound model, the CuS/HA hydrogel can effectively increase the collagen deposition, upregulate the expression of VEGF, and enhance angiogenesis, which might contribute to promote wound healing.

## Data Availability Statement

The raw data supporting the conclusions of this article will be made available by the authors, without undue reservation.

## Ethics Statement

The animal study was reviewed and approved by the Animal Ethical Committee of the West China Hospital of Sichuan University.

## Author Contributions

WZ performed the experiments, writing the manuscript, and the discussion of the results. LZ performed the experiments. YC and CY were involved in the discussion of the results. MT was responsible for conceptualizing, performed the experiments, the discussion of the results, and revising the manuscript.

## Conflict of Interest

The authors declare that the research was conducted in the absence of any commercial or financial relationships that could be construed as a potential conflict of interest.
