# Supplementary material for: Copper Sulfide Nanoparticles-Incorporated Hyaluronic Acid Injectable Hydrogel With Enhanced Angiogenesis to Promote Wound Healing
Source: Front Bioeng Biotechnol. 2020 May 8;8:417. doi: 10.3389/fbioe.2020.00417 (PMC7225278; doi:10.3389/fbioe.2020.00417)
Supplement: Supplementary file 1 [file Image_1.pdf]

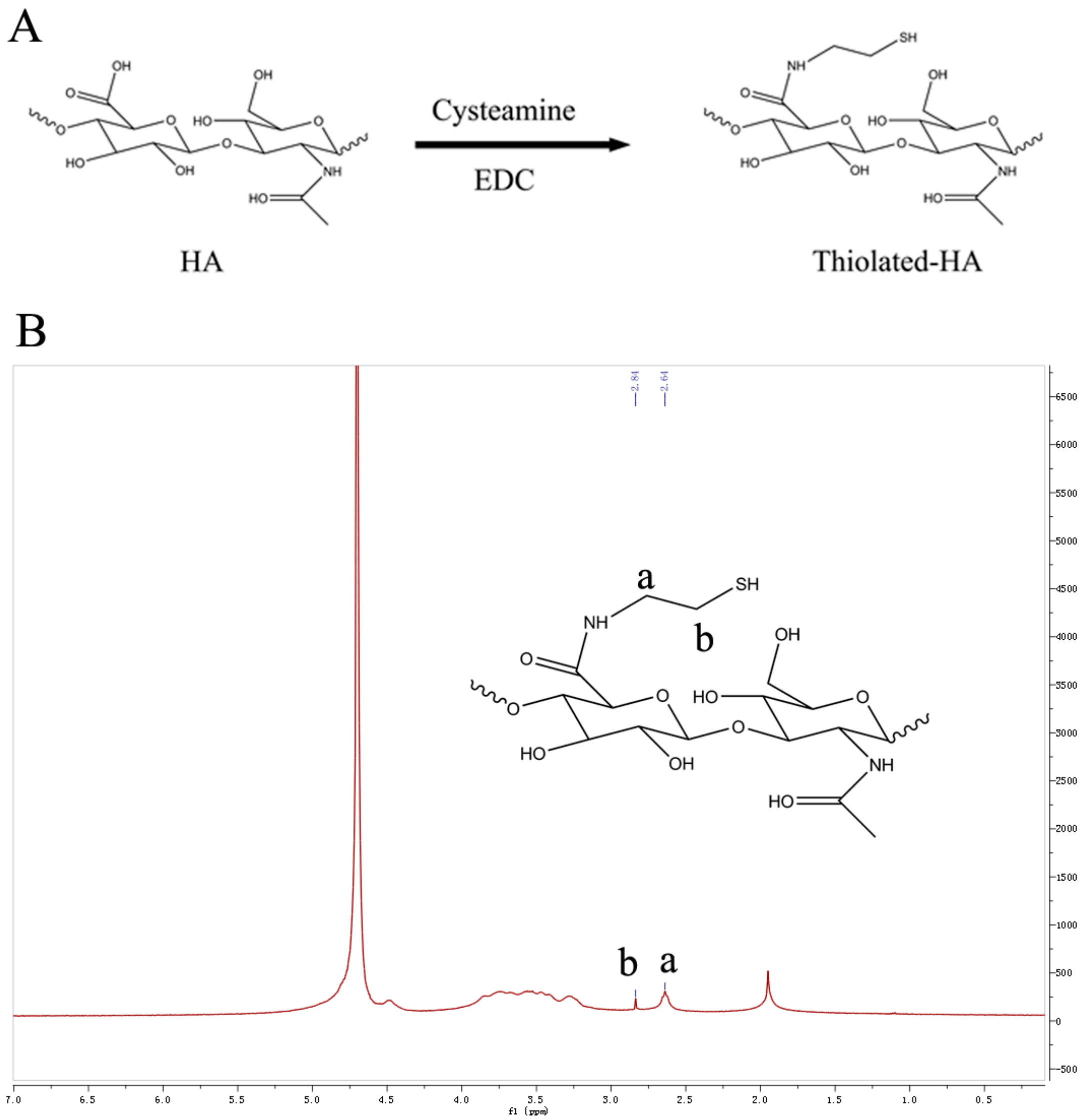

FIGURE S1. (A) The synthesis pathway of the thiolated HA; (B) The ^{1}HNMR of the thiolated HA.

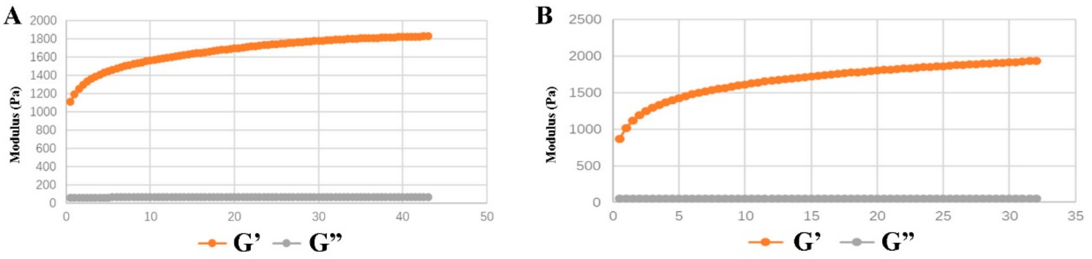

FIGURE S2. The rheological test of the HA hydrogel (A) and CuS/HA hydrogel (B).

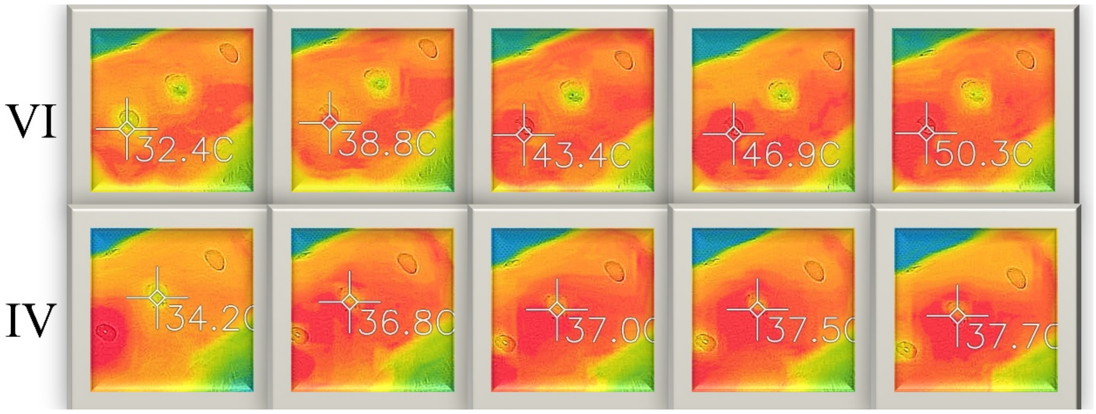

FIGURE S3. The record of the temperature change in mice skin wounds during the phototherapy.

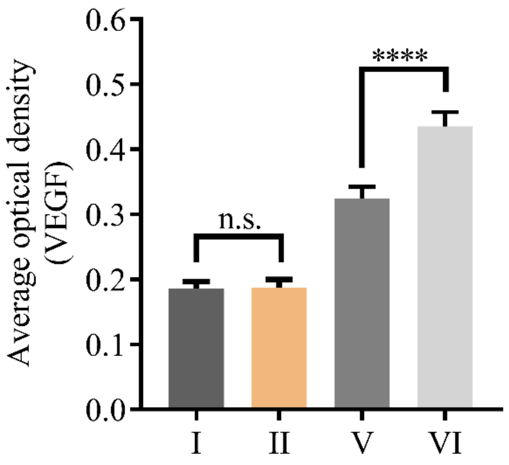

FIGURE S4. VEGF expression in group I, II, V, and VI.
